# Impact of seed color and storage time on the radish seed germination and sprout growth in plasma agriculture

**DOI:** 10.1038/s41598-021-81175-x

**Published:** 2021-01-28

**Authors:** Pankaj Attri, Kenji Ishikawa, Takamasa Okumura, Kazunori Koga, Masaharu Shiratani, Vida Mildaziene

**Affiliations:** 1 Center of Plasma Nano-Interface Engineering, Kyushu University, Fukuoka, 819-0395 Japan; 2 Department of Electronics, Kyushu University, Fukuoka, 819-0395 Japan; 3 Center for Novel Science Initiatives, National Institute of Natural Science, Tokyo, Japan; 4 Faculty of Natural Sciences, Vytautas Magnus University, 44404 Kaunas, Lithuania

**Keywords:** Applied physics, Plasma physics, Mass spectrometry, Plant sciences, Physics

## Abstract

The use of low-temperature plasma for the pre-sowing seed treatment is still in the early stage of research; thus, numerous factors affecting germination percentage, seedling growth, and yield remains unknown. This study aimed to estimate how two critical factors, such as harvest year and seed coat color, affect the percentage of germination and seedling growth after plasma treatment. Radish seeds stored for 2 and 1 year after harvesting (harvested in 2017 and 2018) were sorted into two colors (brown and grey) to investigate the plasma effect on harvest year and seed coat color. We analyzed the amounts of seed phytohormones and antioxidant (γ-tocopherol) were analyzed using mass spectrometry, and physical changes were studied using SEM, EDX, and EPR to understand the mechanism of plasma-induced changes in radish seeds. The obtained results revealed that plasma treatment on seeds affects the germination kinetics, and the maximal germination percentage depends on seed color and the time of seed storage after harvest. Through this study, for the first time, we demonstrated that physical and chemical changes in radish seeds after plasma treatment depends upon the seed color and harvest year. Positive effects of plasma treatment on growth are stronger for sprouts from seeds harvested in 2017 than in 2018. The plasma treatment effect on the sprouts germinated from grey seeds effect was stronger than sprouts from brown radish seeds. The amounts of gibberellin A3 and abscisic acid in control seeds strongly depended on the seed color, and plasma induced changes were better in grey seeds harvested in 2017. Therefore, this study reveals that Air scalar-DBD plasma's reactive oxygen and nitrogen species (RONS) can efficiently accelerate germination and growth in older seeds.

## Introduction

Sprouts have long been consumed globally due to their high nutritional value^1^. Brassica sprouts, in particular broccoli (*Brassica oleracea* var. *italica*) and radish (*Raphanus sativus* L.) sprouts, contain substantial amounts of antioxidants, vitamin C and health-promoting compounds such as glucosinolates and phenolic compounds^2,3^. It was shown that radish sprouts have anticancer and antioxidant activities both in vivo and in vitro^4,5^*.* To consider the health benefits of radish sprouts, it is important to increase radish sprouts production^6^. Besides other methods^7–10^, different (chemical, physical, and biological) pre-sowing seed treatments can be applied for this purpose. The physical seed treatment, including electromagnetic waves, ionizing radiations, cold atmospheric plasma (CAP), etc.^9,10^, were used to accelerate the rate of seed germination, enhance plant growth, and increase agricultural yields. Recently, the use of low-temperature plasma to treat seeds has increased frequently^10^. For example, seeds can be treated with plasma at low temperatures and in less harsh conditions than conventional treatments. However, the germination rate mechanism, germination percentage, and seedling growth using plasma technology are still unknown.

In previous studies, growth enhancement and increased root-to-shoot mass ratio of radish sprouts were observed after seed treatment with atmospheric pressure plasma (APP)^11–13^. We assumed that plasma generated reactive oxygen and nitrogen species (RONS) may reach the embryo or endosperm through the seed coat, enhancing the plant growth. EPR (electron paramagnetic resonance) spectroscopic method was used to detect the paramagnetic species such as Fe^3+^, organic free radicals, and Mn^2+^in various plant seeds^14,15^. We also observed that scalar dielectric barrier discharge (DBD) treatment of radish seeds increase organic free radicals' intensity for grey but not for brown seeds^16^. However, the effects of plasma conditions, harvest year, and seed coat color on the paramagnetic species like Fe^3+^ and Mn^2+^ of radish sprouts are still unknown.

In this study, we have compared the effects of RONS produced from Air scalar-DBD plasma treatment on germination rate and early growth of radish seeds harvested in two different years (2017 and 2018), and each seed lot was sorted into two groups (brown and grey) by color. We investigated the RONS-induced changes in physical, chemical, phytohormones, and antioxidant levels in radish seeds before and after plasma treatment.

## Result and discussion

### Germination rate and seedling growth

**Figure 1:**
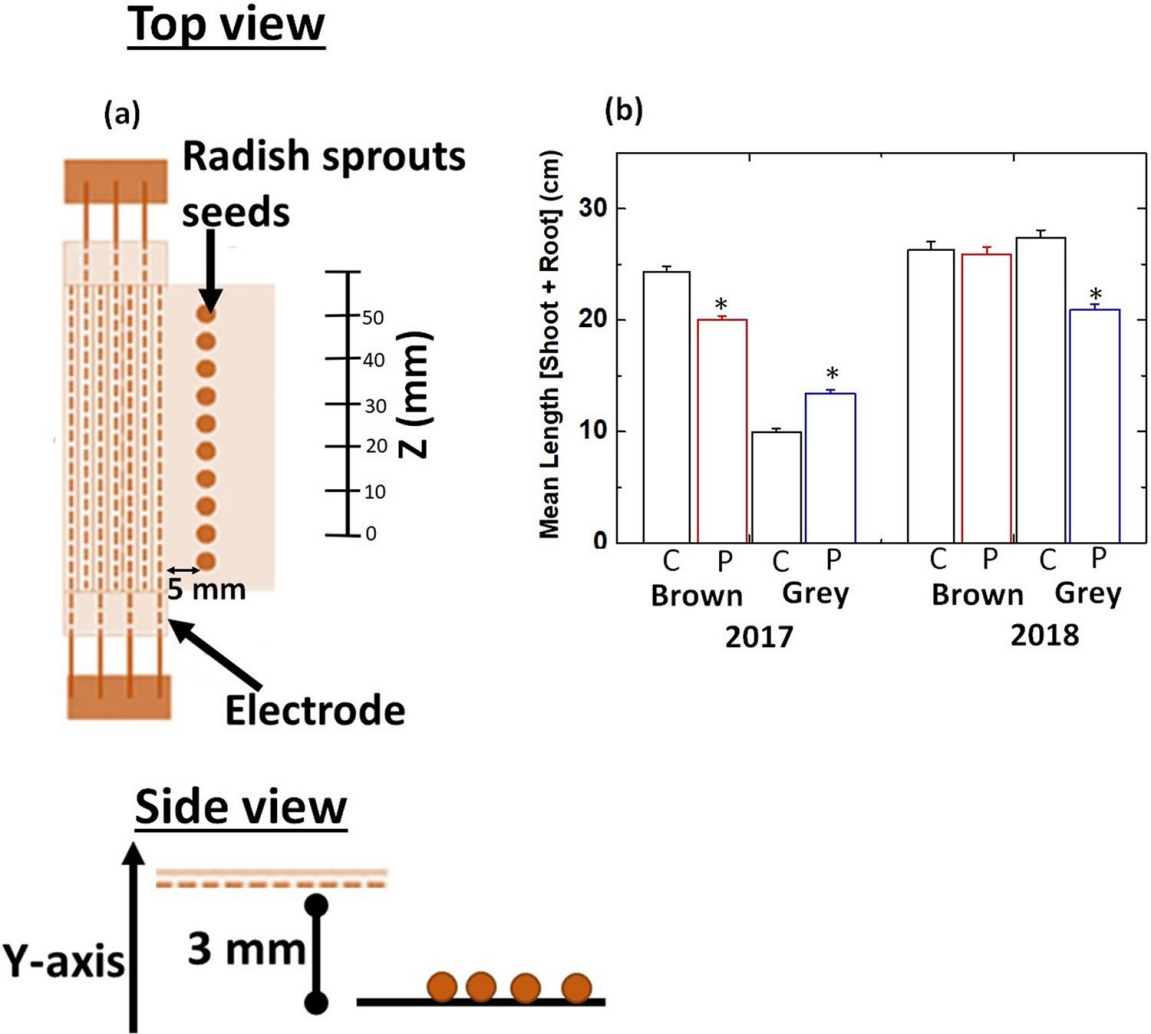
**(a)** Schematic display of radish seed position during plasma treatment (top and side view); **(b)** mean length of sprouts (shoot + root) germinated from control (C) and plasma treated (P) radish seeds harvested in 2017 and 2018 year. Results are presented as means ± SEM (n = 40).*Statistically significant difference in comparison to control (p ≤ 0.05).

The experiment was performed in 2019; however, radish seeds were stored at 4 °C for 2 years (harvested 2017) and 1 year (harvested in 2018). DBD equipment used for seed treatment was described in our previous article and material and method Sect. ^11^ Ten seeds of radish were placed at a distance of 5 mm apart from the electrode edge of Air scalar-DBD and 3 mm below the electrode, as shown in Fig. 1a. The details of seed treatment was explained in our earlier work^12^. The average seedling length was maximized with 3 min of plasma irradiation.

**Figure 2:**
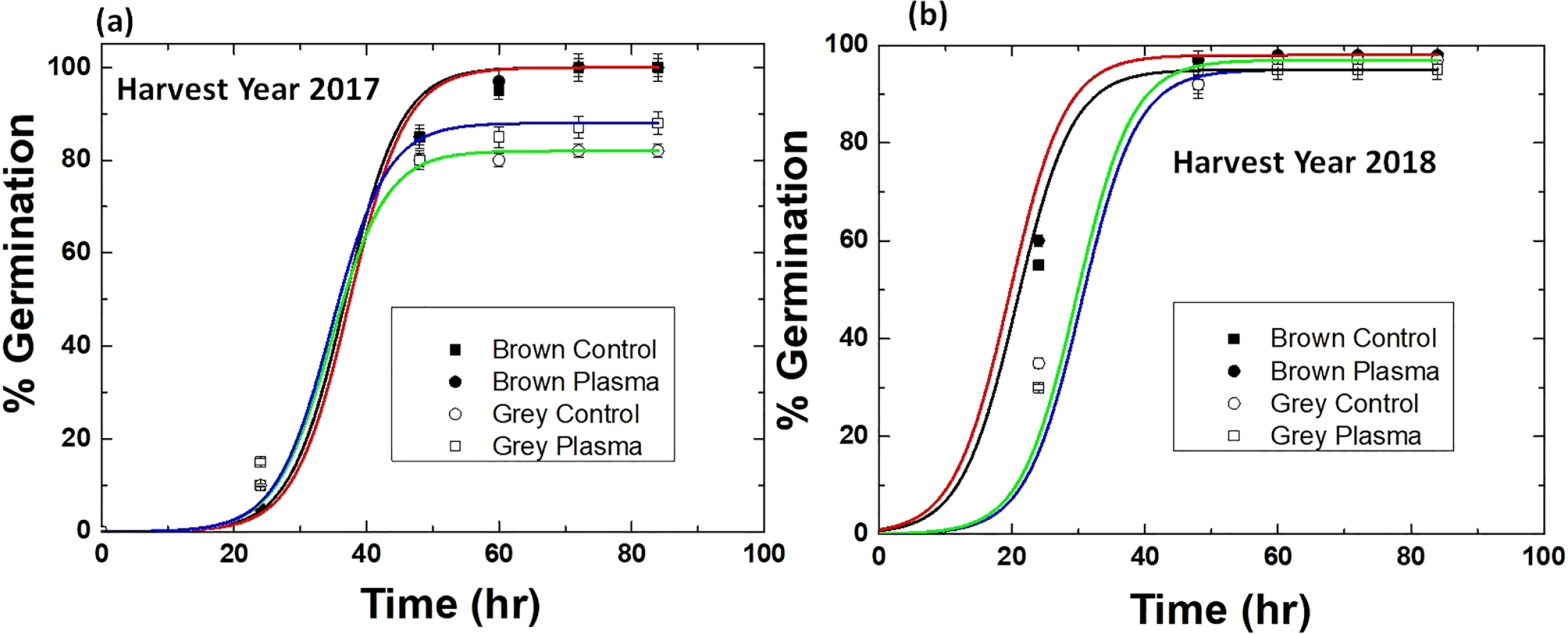
Germination kinetics of brown and grey radish seeds harvested in 2017 **(a)** and 2018 **(b)**. The points are fitted using Boltzmann non-linear fit. Brown control (black line), brown plasma (red line), grey control (green line), and grey plasma (blue line).

A separate experiment was performed to detect the optimal treatment time. For this, we treated 2017 harvest year radish seeds for 3, 9, and 30 min with plasma. For brown and grey seeds harvested in 2017, an increase of plasma treatment duration from 3 to 9 min or 30 min reduced the germination percentage (by 10% and 20–22%, respectively, compared to control) [Figure S1, supporting information]. Since longer treatment durations (9 and 30 min) showed a negative effect on germination. Therefore, for further experiments, we used 3 min of plasma seed treatment. The obtained results indicate that germination kinetics, the maximal germination percentage, and the effects of plasma treatment on radish seed germination depended on seed color and seed storage time after harvest. Figures 2 and S1, show that for control seeds of both brown and grey color harvested in 2018, 100% maximal germination percentage was achieved, although brown seeds germinated more rapidly (50% of seeds germinated 20 h after imbibition) compared to grey seeds (50% of seeds germinated 32 h after imbibition). Maximal germination of control grey seeds harvested in 2017 was significantly lower (by 18%) compared to brown seeds. Plasma treatment for 3 min increased the maximal germination percentage by 8% for grey but not for brown seeds germinated in 2017 (Figs. 2 and S2).

Figures 1b and S2 show the change in the total length of sprouts (root + shoot) before and after plasma treatment for all treatment conditions. The average length of brown seed sprouts was 24 ± 0.5 cm for 2017 harvest, whereas, after the plasma treatment, it was 20 ± 0.4 cm (Fig. 1b) on the 4th after treatment. The average length of sprouts from brown seeds for 2018 harvest not changed significantly after plasma treatment (Fig. 1b). The average length of sprouts from grey seeds in control was 10 ± 0.3 and 27 ± 0.7 cm for 2017 and 2018 harvest year, respectively. Plasma treatment increased the length of sprouts from grey seeds by 30%in 2017 harvest group and decreased by 22% in 2018 harvest group (Fig. 1b).

**Figure 3:**
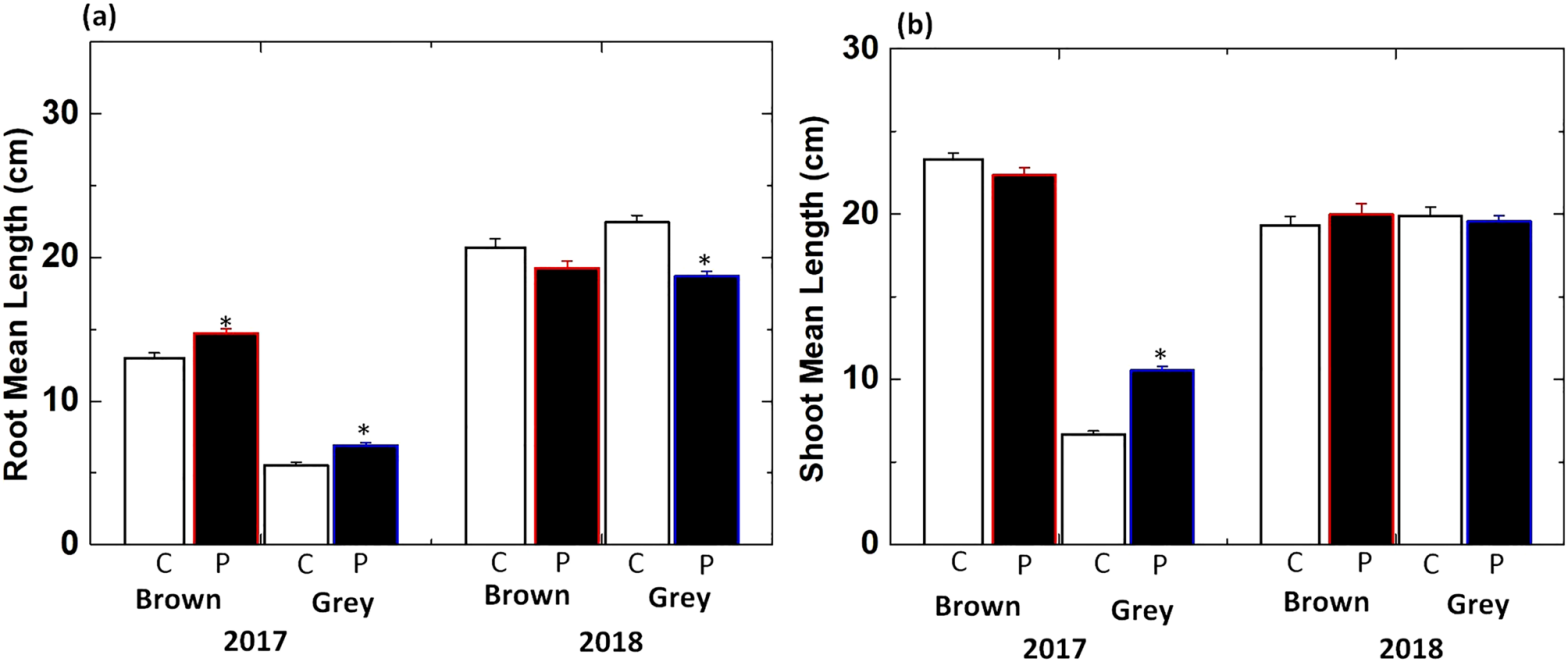
Average length of **(a)** roots and **(b)** shoots of sprouts germinated from control (C) and plasma treated (P) radish seeds harvested in 2017 and 2018 year. Results are presented as means ± SEM (n = 40).*Significantly different from the control group (p < 0.05).

Figure 3a,b show the change in root and shoot length of germinated radish sprouts induced by seed treatment with DBD. In control of the 2017-harvest year, the roots and shoots of sprouts germinated from brown seeds were longer (by 50 and 70%, respectively) compared to those from grey seeds. The length of shoot and root can vary depending on the year of seed harvest and seed color. Plasma treatment improved the growth of roots of radish sprouts for the 2017-harvest year, but an increase in root length was larger in seedlings from grey seeds (25%) compared to those from brown seeds (13%). Plasma treatment induced 7% decrease in root length of sprouts from brown seeds harvested in 2018 and decreased root length by 16% in seedlings from grey seeds (Fig. 3a). We observed decreased shoot length (4%) in sprouts of brown seeds, and a significant increase (59%) for grey seeds after plasma treatment was observed in the 2017 harvest groups (Fig. 3b). However, shoot length was not changed significantly after plasma treatment for sprouts from brown seeds and grey seeds harvested in 2018. The morphometric analysis showed that positive effects of plasma treatment on growth are stronger for sprouts from seeds harvested in 2017 than in 2018. The development of sprouts germinated from grey seeds is affected stronger than sprouts from brown radish seeds.

### EPR (electron paramagnetic resonance) spectroscopy of seeds before and after plasma treatment

**Figure 4:**
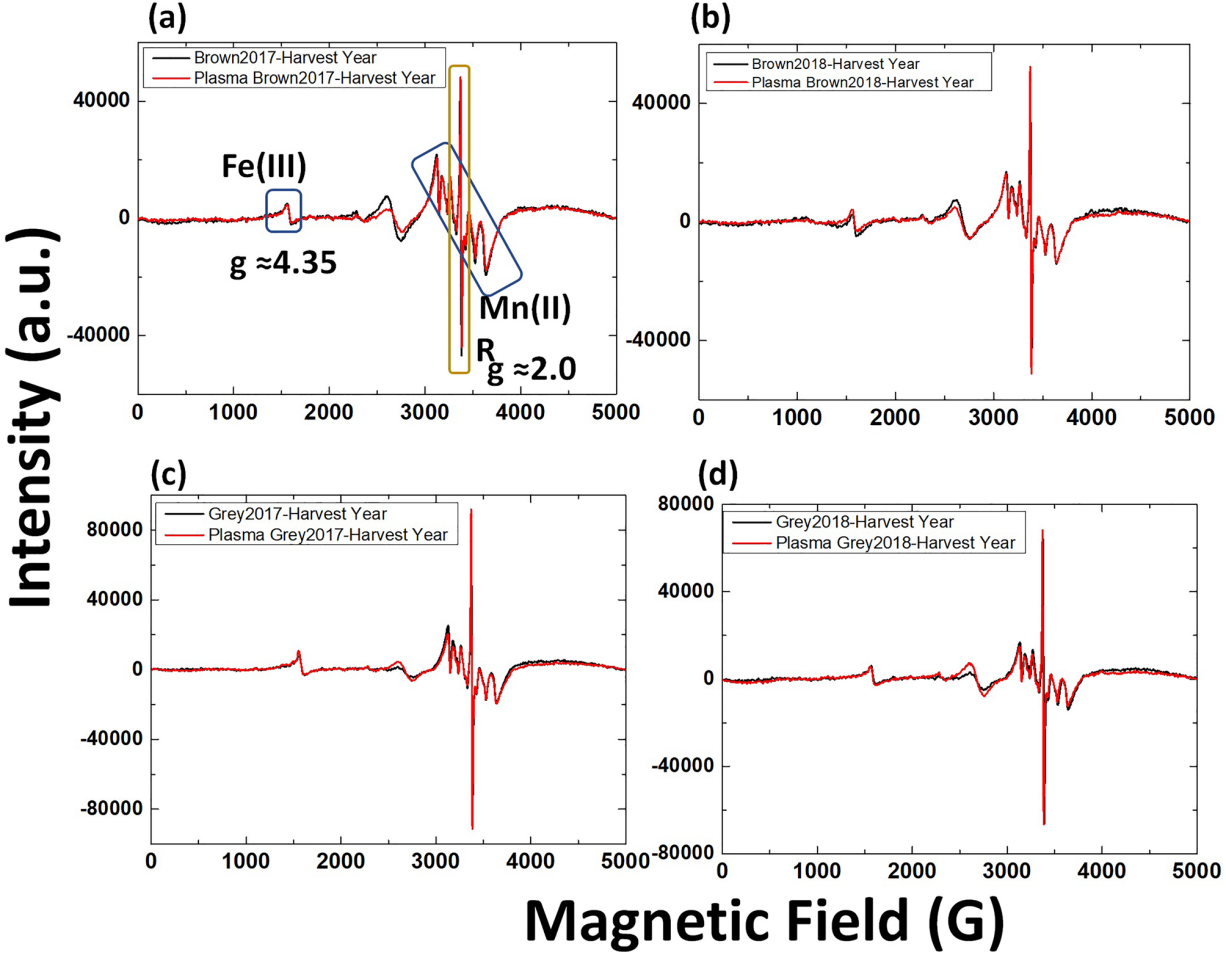
EPR spectra of radish seeds harvested in 2017 or 2018 before (black line) and after plasma treatment (red line) **(a)** brown seeds harvested in 2017, **(b)** brown seeds harvested in 2018, **(c)** 2017-harvest year grey seeds and **(d)** 2018-harvest year grey seeds. Ten seeds were used in one measurement.

Figure 4 shows the EPR spectrum of radish sprout seeds. The EPR spectra contain six hyperfine lines centered around g = 2 recognized as Mn^2+^ complexes in slightly distorted octahedral symmetry^17–19^. The sharp and intense line at g = 2.0 may be attributed to the aromatic organic radical related to the semiquinone radical^20–22^. The relatively weak broad shoulder at g = 4.35 originated to the low field side of the Mn^2+^ sextet from a Fe^3+^ paramagnetic center. The components of the spectrum are similar to the previously reported structures^23–25^. The peak at g = 4.35 related to high spin Fe^3+^ complexes with rhombically distorted symmetry^19,26^.

**Figure 5:**
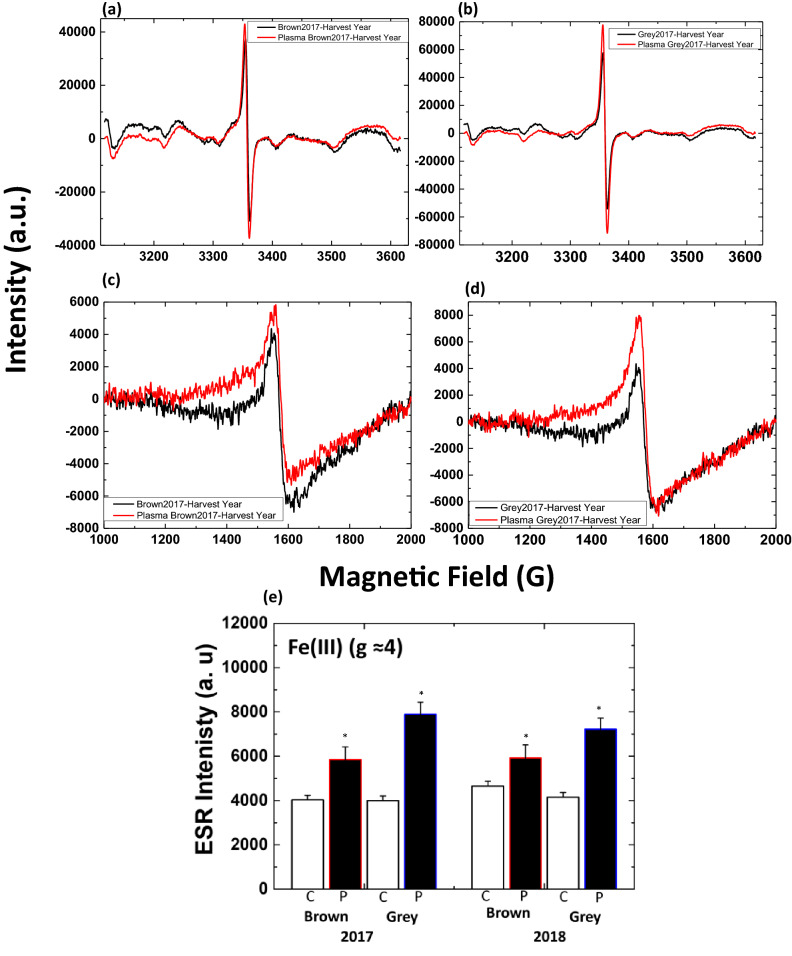
EPR spectra for radish seeds harvested in 2017-at **(a)** g ≈ 2 brown seeds, **(b)** g ≈ 2 grey seeds, **(c)** g ≈ 4 brown seeds, **(d)** g ≈ 4 grey seeds before (black line) and after plasma treatment (red line), **(e)** EPR intensity at g ≈ 4, before and after plasma treatment.*Statistically significant difference from the control group (p < 0.05).

Treatment of radish seeds with RONS produced from plasma did not lead to the appearance of new EPR peaks but led to the increased intensity of the observed EPR peaks (Fig. 5). For brown and grey seeds harvested in 2017, EPR peak intensity for Mn^2+^ increases after the plasma treatment with respect to the control (Fig. 5a,b). However, the increase in intensity in grey seeds after plasma treatment was higher compared to that in brown seeds (Fig. 5a,b). Similar behavior was observed for 2018-harvest brown and grey seeds EPR intensity after plasma treatment (Figure S3). Fe^3+^peaks at g ≈ 4 for brown and grey seeds (2017 and 2018-harvest years) also increased after plasma treatment (Fig. 5c–e). A more significant increase in intensity was observed for the grey seeds after plasma treatment than for brown seeds.

### Phytohormones and antioxidant level in seeds before and after plasma treatment

We measured the plasma treatment effects on the amounts of two phytohormones gibberellin A3 (GA3), abscisic acid (ABA) (main phytohormones involved in the control of seed germination^27^) and antioxidant γ-tocopherol (which is the main form of vitamin E in seeds^28^) in radish sprout seeds. For this, we crushed the seeds and extracted phytohormones in methanol (99.9%) for 24 h at 4 °C (details are described below). The obtained supernatant was used for mass spectrometry analysis to detect the concentration of GA3, ABA, and γ-tocopherol.

The obtained results showed that the amounts of GA3 and ABA in control seeds were strongly dependent on the seed color (Table 1). Particularly large difference was in seeds harvested in 2017: the amount of GA3 in brown seeds was 2.8 times larger, and the amount of ABA was 3.5 times smaller compared to the corresponding amounts in control grey seeds. These findings indicate that the decreased germination capacity of grey seeds (2017) (Fig. 2) compared to all other seed groups may be explained by their highest content of the germination inhibitor ABA and the lowest GA3/ABA ratio (9.5 times smaller in grey seeds compared to the brown ones). Differences in the content of phytohormones were less strong for seeds of different colors harvested in 2018. The amount of ABA was by 27% higher, and the amount of gibberellin A3 was 2 times smaller (GA3/ABA ratio 2.6 times smaller) in grey seeds than brown seeds. Thus, the same color-dependent tendencies were displayed as between grey and brown seeds harvested in 2017.

**Table 1:** The amount of GA3, ABA, GA3/ABA, and γ-tocopherol in control and plasma treated brown and grey radish sprout seeds harvested in 2017 and 2018.

	GA3, ng/g	ABA, ng/g	GA3/ABA	Tocopherol, μg/g
**Control 2017 harvest seeds**				
Brown	1.23 ± 0.03(0.08)	2.19 ± 0.14(0.35)	0.57 ± 0.04(0.09)^**$$**^	1.71 ± 0.06(0.15)^$$^
Grey	0.44 ± 0.02(0.05)^**#**^	7.65 ± 0.11(0.23)^**##**^	0.06 ± 0.003(0.007)^##^	2.25 ± 0.04(0.11)
**Plasma treated seeds 2017 harvest**				
Brown	1.3 ± 0.07(0.18)	1.79 ± 0.02(0.05)*	0.73 ± 0.05(0.11)*	1.67 ± 0.04(0.09)
Grey	1.25 ± 0.04(0.10)**	4.07 ± 0.10(0.24)**	0.31 ± 0.01(0.02)**	2.04 ± 0.04(0.10)*
**Control 2018 harvest**				
Brown	1.22 ± 0.06(0.15)	4.06 ± 0.24(0.59)	0.31 ± 0.02(0.04)	2.58 ± 0.05(0.12)
Grey	0.60 ± 0.03(0.80)^#^	5.55 ± 0.16(0.41^**)##**^	0.12 ± 0.01(0.02)^**##**^	2.39 ± 0.03(0.07)^#^
**Plasma treated seeds 2018 harvest**				
Brown	1.88 ± 0.19(0.47)	1.84 ± 0.04(0.09)**	1.03 ± 0.12(0.30)**	2.47 ± 0.03(0.08)
Grey	0.71 ± 0.04(0.11)	5.94 ± 0.17(0.43)	0.11 ± 0.01(0.02)	2.38 ± 0.02(0.05)

Results are presented as means ± SEM(SD), n = 6.

*Statistically significant effect of CP (p < 0.05).

**Statistically significant effect of plasma (p < 0.001).

^$^Statistically significant effect of harvest year for the same color (p < 0.05).

^$$^Statistically significant effect of harvest year for the same color (p < 0.001).

^#^Statistically significant effect of color for the same harvest year(p < 0.05).

^##^Statistically significant effect of color for the same harvest year (p < 0.001).

Plasma treatment did not change the amount of GA3 in brown seeds of the 2017 harvest. However, in grey seeds treated with plasma GA3 amount increased 2.8 times (Table 1). The amount of ABA was reduced after plasma treatment much stronger (47%) in grey seeds than brown seeds (18%) harvested in 2017. Plasma induced increase of GA3/ABA ratio was in grey seeds (5.2 times) was also remarkably larger as compared to the growth observed in the brown seeds (28%) (Table 1). Changes in the content of phytohormones induced by plasma treatment in seeds harvested in 2018 were smaller than the respective changes in older (2017) seeds. Changes in the ratio of GA3/ABA was statistically insignificant both for grey seeds but significant for brown seeds (2018 harvest). Thus, the largest increase in the GA3 to ABA ratio was observed in grey seeds harvested in 2017 after plasma treatment. Since ABA is an inhibitor of germination and GA stimulates germination^27^, the observed stimulation of germination in grey seeds (2017) after plasma treatment (Fig. 2a) is in good agreement with changes in the amounts of phytohormones.

The amount of γ- tocopherol in radish sprout seeds was considerably higher in comparison to amounts of phytohormones (Table 1). The brown seeds harvested in 2018 were 50% higher compared to that in brown seeds harvested in 2017, indicating that antioxidant amount decreased in seeds during storage due to aging. However, the γ- tocopherol amount was approximately the same in grey seeds harvested in 2018 and 2017. Plasma did not change significantly γ- tocopherol amount in brown seeds harvested both in 2017 and in 2018, as well as in grey seeds harvested in 2018. However, the γ- tocopherol amount decreased by 10% in 2017-harvest year grey seeds, indicating possible oxidative damage induced by plasma treatment. Thus, older grey seeds (2017) showed larger changes in the amounts of GA3, ABA, and γ- tocopherol after plasma treatment compared to other seed groups. The changes in phytohormones in grey seeds (2017-harvest) are consistent with the previously reported work^29^. The content of GA increases and ABA decreases, and their correlation with plasma effects on germination in freshly harvested seeds^29^.

### SEM and EDX analysis of grey seeds before and after plasma treatment

**Figure 6:**
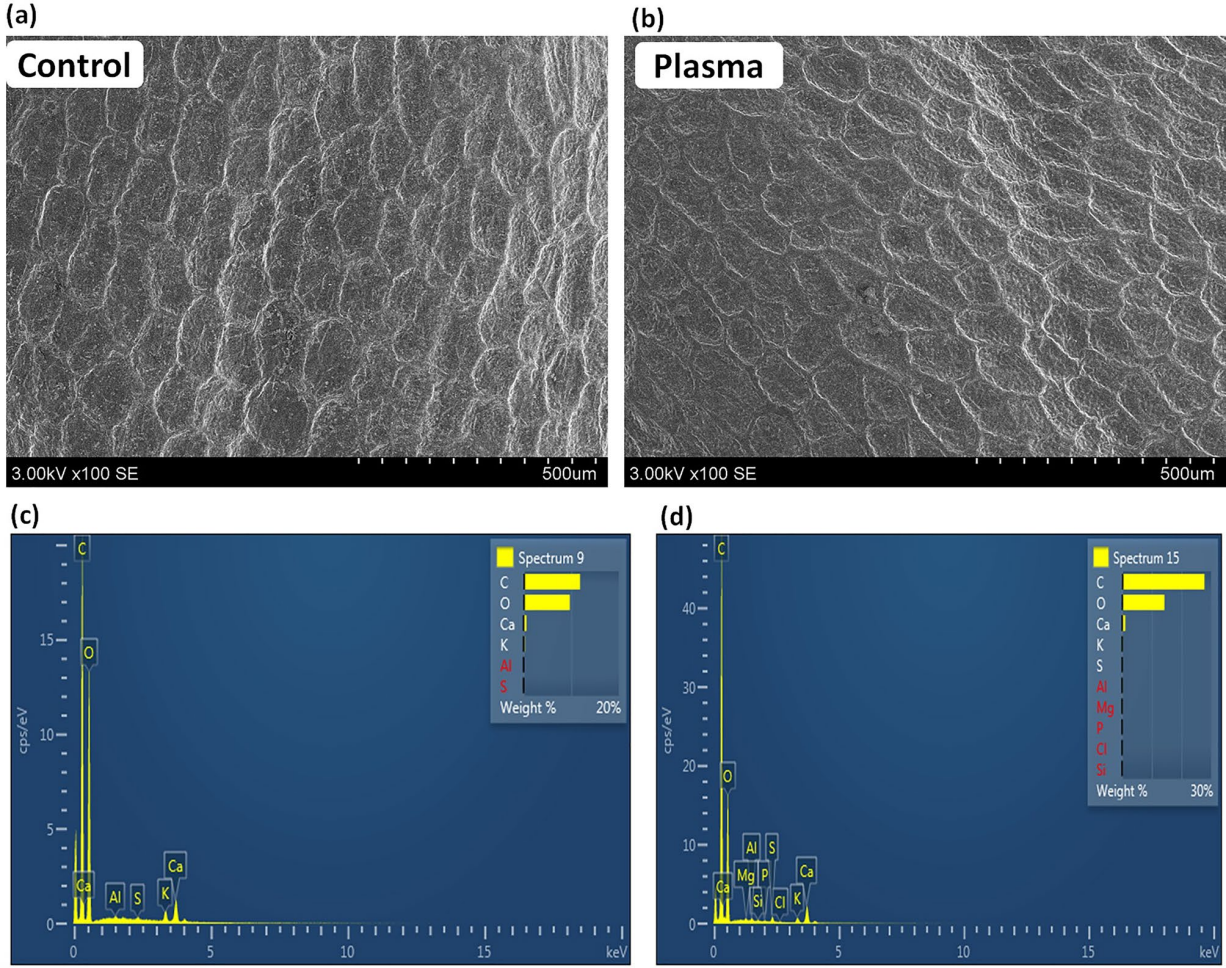
SEM image of radish seeds **(a)** control; **(b)** after plasma treatment; EDX spectra of **(c)** grey seed control and **(d)** after plasma treatment.

Among all the radish seed samples, 2017-harvest grey seeds displayed significant changes in phytohormone amounts, germination, and sprout growth after plasma treatment (Figs. 1–3 and Table 1). Therefore, to explore the physical changes in grey seeds after plasma treatment, we performed the SEM and EDX analysis (details are described below). Figure 6a,b, and Figure S4 show the SEM images representing the modification of the radish seed surface structure after DBD plasma treatment. It seems that after plasma treatment, there were no significant changes on the seed surface. EDX analysis provides the elemental mapping of grey radish sprouts seeds (2017-harvest year) in a thickness of microns before and after plasma treatment (Fig. 6c,d, Table 2).

**Table 2:** EDX analysis of control and plasma treated grey seeds.

Content	Wt % for control grey seeds	Wt % for plasma-treated grey seeds
C	11.85	27.73
O	9.68	14.23
Mg	ND	0.05
Al	0.03	0.06
Si	ND	0.02
P	ND	0.03
S	0.03	0.17
Cl	ND	0.03
K	0.24	0.26
Ca	0.58	1.06

*ND* not detected.

The EDX analysis in Table 2, shows the increase in all contents like C, O, Mg, Al, Si, P, S, Cl, K, and Ca after the plasma treatment. A previous study reported the change in S and P distribution in nasturtium seeds after plasma treatments using EDX^30^. However, in the present study P signals are not observed in control of grey seeds (2017-harvest), whereas P signals have appeared after the plasma treatment.

On the other hand, S signals are present in both control and plasma-treated grey seeds; however, the S content percentage increased after the plasma treatment. In general, the S and P are present in the embryonic axis^31^, so plasma treatment results in deeper etching on the surface of seed coat layers that revealed with different elemental composition and possibility to increase the concentration of S and P. The primary source of S is the defense compounds like S-rich proteins, glutathione, hydrogen sulfide, phytochelatins, etc., which are crucial for plant survival^32^. Additionally, the phytomelanin pigments of seeds containing S compounds might contribute to increased S concentration^33^ after plasma treatment, as plant melanins react with strong oxidizing agents produced during plasma treatment, like H_2_O_2_, O_3_, O_2_^•‾^, OH, HO_2_, etc.

P presence in the seeds help in seedling growth and germination, and P mainly occurs in phytate form in seeds around 30–80%^34^. The phytate occurs in seeds as mixed salts with cations (K, Mg, Ca, Mn, Fe, and Zn), and hydrolysis of phytate is a source of P for developing seedlings^35^. The correlation between P and bio-metallic cations such as Fe, Mn, K, Zn, Cu, and Mg, was reported previously^35,36^. Hence, the detection of Fe and Mn signals using EPR may be due to the phytate complex. After the plasma treatment, the hydrolysis of phytate or phytate complex is possible to break down, or the number of free radical increases, which results in the release of cations and anions. That might result in the detection of various elemental compositions after plasma treatment.

## Conclusion

We concluded that the plasma treatment of seeds accelerates the germination process by modification of the physical and biochemical components in seeds. Changes induced in seed coat EPR signals reveal the differences in organic free radicals and metal ions after plasma treatment. In line with the previous reports^29,37–39^, plasma treatment changed the amounts of phytohormones in seeds, and the induced changes correlated with the effects on germination; however, in this study, the dependence of such plasma effects on seed color was demonstrated for the first time. Hence, plasma treatment provides unique physical and biochemical modifications that increase germination percentage and stimulation of seedling growth for longer-stored seeds. This study speculates that plasma treatment can initiate the chemical reaction in long stored seeds that improves their germination and seedling growth. Thereby, plasma treatment can solve the germination and growth problem in older seeds.

## Material and methods

### Experimental setup

Scalar dielectric barrier discharge (DBD) device consisted of 20 stainless rod electrodes of 1 mm in outer diameter and 60 mm in length covered with a ceramic tube of 2 mm in outer diameter. The electrodes are arranged parallel with a spacing of 0.2 mm. DBD plasma discharge was generated between the electrodes by supplying a 14.4 kHz AC high voltage (Logy Electric, LHV-09K). The discharge power density in the Air was 3.05 W cm^−2^. The relative humidity was 40–60%. The distance between the electrode and seeds was 5 mm, and the plasma irradiation time was 3 min.

### Seed treatment and germination

We used a scalable DBD device for plasma irradiation of radish (*Raphanus sativus* L.) sprout seeds. Seeds of radish sprouts were bought from Nakahara Seed Co., Japan, in 2017 and 2018. The control and plasma treated radish seeds (3 replicates × 4 conditions × 40 seeds = 480 seeds in total) were used for experiments. Ten radish seeds were treated at once for 3 min. After treatment, the seeds were placed on the container with deionized water for germination, as shown in Figure S5a. The container with seeds was kept in the dark incubator, as shown in Figure S5b, for 84 h at 25 °C temperature. The number of germinated seeds was counted 5 times (24, 48, 60, 72, and 84 h after imbibition). On 4th day the morphometric analysis of sprouts was performed, measuring the length of shoots and roots for all 4 experimental groups.

### EPR measurements

EPR measurements were performed with ten control seeds (for both 2017 and 2018 harvest and of brown and grey color), and later these seeds were treated with DBD for 3-min. The variable temperature unit was employed to change the temperature in the EPR cavity. The measurement was performed at 113 K using liquid nitrogen. For the EPR measurements, the power of the X-band microwave and modulation frequency was 2.15 mW and 100 kHz, respectively. The magnetic field range was from 0 to 5000 Gauss. The spectra of barley seeds were recorded in quartz sample tubes and spectrum simulations using Bruker’s Win EPR Simfonia software. The measurements of EPR spectra were performed 3 times with 6 replicates to reduce the error, and the final spectra are average spectra.

### Sample preparation for mass spectrometry

Ten radish seeds were added in the 2 ml round bottom tube with a stainless bead and pulverized at 2600 rpm for 3 min. Further, add the methanol (99.9%) to the 2 ml round bottom tube and kept for 24 h at − 20 °C, and the obtained suspension was centrifuged (Eppendorf centrifuge 5430 R) at 3000 rpm at 4 °C for 5 min. The supernatant was transferred into the tube, filtered using 0.22 μm filter, and centrifuged again at 4 °C for 5 min. The obtained supernatant was used for mass spectrometry analysis.

### Mass spectrometry

Mass spectrometry measurement was performed with LC (Agilent; G7116A) and triple quadrupole mass spectrometer (Agilent; G6470A). A 4.6 × 100 mm i.d. Zorbax column (Agilent; XFB-C18) was used at a 1 mL/min flow rate. The LC gradient was methanol in 5 mM ammonia acetate/H_2_O as follows: 20 to 100% in 5 min. Conditions for MS/MS analysis of each LC peak included a capillary voltage of 4000 V, nozzle voltage of 500 V, a nebulizing pressure of 45 psi, and the sheath gas temperature at 375 °C with 11 L/min of low rate.

### SEM and EDX analysis

Scanning electron microscopy (SEM) images using Hitachi S-3400N.The energy-dispersive X-ray spectroscopy (EDX) mapping was done using an energy-dispersive X-ray analyzer (X-Max50, Oxford Instruments, Abingdon-on-Thames, British) at an accelerating voltage of15 kV.

## Supplementary Information

Supplementary Figures.
