# Supplementary material for: Impact of seed color and storage time on the radish seed germination and sprout growth in plasma agriculture
Source: Sci Rep. 2021 Jan 28;11:2539. doi: 10.1038/s41598-021-81175-x (PMC7844220; doi:10.1038/s41598-021-81175-x)
Supplement: Supplementary file 1 — Supplementary Figures. [file 41598_2021_81175_MOESM1_ESM.docx]

**Supporting information**

**Impact of seed color and storage time on the radish seed germination and sprout growth in plasma agriculture**

Pankaj Attri^1^*, Kenji Ishikawa^1^, Takamasa Okumura^2,3^, Kazunori Koga^2,3^, Masaharu Shiratani^1,2^, and Vida Mildaziene^4^

^1^ Center of Plasma Nano-interface Engineering, Kyushu University, Fukuoka 819-0395, Japan

^2^ Department of Electronics, Kyushu University, Fukuoka 819-0395, Japan

^3^ Center for Novel Science Initiatives, National Institute of Natural Science, Japan

^4^Faculty of Natural Sciences, Vytautas Magnus University, Kaunas LT-44404, Lithuania

**Figure Captions for supporting files**

**Figure S1:** Change in germination percentage of brown and grey radish seeds harvested in 2017 after different types of treatment. The final germination was counted on the 4^th^ day after imbibition. The results are presented as means percentage ±SEM (n= 40).

**Figure S2:** Radish sprouts germinated from brown and grey seeds harvested in 2017 and 2018.

**Figure S3:** (a) EPR spectra at g =2 for brown seeds harvested in 2018 before (black) and after (red) plasma treatment and (b) EPR spectra at g =2 for grey seeds harvested in 2018 before (black) and after (red) plasma treatment.

**Figure S4:** SEM image of grey seed (a) control; (b) after plasma treatment for 2017-HY.

**Figure S5:** (a) Picture radish seeds of placed in a container for germination**;** (b) the he containers with seeds placed in an incubator where seeds were left to germinate in dark for 4 days.

**Figure S1**

**Figure S2**

**Figure S3**

**Figure S4**

**Figure S5**
